# A review of economic evaluation models for cardiac resynchronization therapy with implantable cardioverter defibrillators in patients with heart failure

**DOI:** 10.1007/s10198-015-0752-3

**Published:** 2016-01-04

**Authors:** F. Tomini, F. Prinzen, A. D. I. van Asselt

**Affiliations:** 1 Department of Clinical Epidemiology and Medical Technology Assessment, Maastricht University Medical Centre, Maastricht, The Netherlands; 2 Department of Physiology, Cardiovascular Research Institute Maastricht (CARIM), Maastricht, The Netherlands; 3 Department of Pharmacy, University of Groningen, Groningen, The Netherlands; 4 Department of Epidemiology, University Medical Centre Groningen, Groningen, The Netherlands

**Keywords:** Review, Cost-effectiveness, Cardiac resynchronization therapy, Cardiac pacing, Implantable cardioverter-defibrillator, Markov chains, Models, Economic, Heart failure, Sudden cardiac death, C63, D61, I18, H43

## Abstract

**Objectives:**

Cardiac resynchronization therapy with a biventricular pacemaker (CRT-P) is an effective treatment for dyssynchronous heart failure (DHF). Adding an implantable cardioverter defibrillator (CRT-D) may further reduce the risk of sudden cardiac death (SCD). However, if the majority of patients do not require shock therapy, the cost-effectiveness ratio of CRT-D compared to CRT-P may be high. The objective of this study was to systematically review decision models evaluating the cost-effectiveness of CRT-D for patients with DHF, compare the structure and inputs of these models and identify the main factors influencing the ICERs for CRT-D.

**Methods:**

A comprehensive search strategy of Medline (Ovid), Embase (Ovid) and EconLit identified eight cost-effectiveness models evaluating CRT-D against optimal pharmacological therapy (OPT) and/or CRT-P.

**Results:**

The selected economic studies differed in terms of model structure, treatment path, time horizons, and sources of efficacy data. CRT-D was found cost-effective when compared to OPT but its cost-effectiveness became questionable when compared to CRT-P.

**Conclusions:**

Cost-effectiveness of CRT-D may increase depending on improvement of all-cause mortality rates and HF mortality rates in patients who receive CRT-D, costs of the device, and battery life. In particular, future studies need to investigate longer-term mortality rates and identify CRT-P patients that will gain the most, in terms of life expectancy, from being treated with a CRT-D.

## Introduction

Cardiac resynchronization therapy (CRT) either via a pacing device (CRT-P) or a pacemaker-defibrillator device (CRT-D) is considered an effective treatment for patients with congestive heart failure (CHF) and disturbances in heart rhythm (arrhythmias) having New York Heart Association (NYHA) class II, III and IV symptoms. Clinical trials have shown that CRT may decrease the risk of death from any cause for CHF patients by 24 % during a mean follow-up time of 16 months (COMPANION study, [1]) to 36 % during a mean follow-up of 29.4 months (CARE-HF study [2]). The addition of an implantable cardioverter defibrillator to the resynchronization therapy (CRT-D) can further reduce the risk of death from any cause by more than 8 % (compared to CRT-P) [3], while the risk of sudden death (SCD) can be reduced by 23 % [4–6]. However, overall costs of CRT-D are high and it is reported that about 25 to 35 % of the patients do not respond to CRT-P [7] while implantable cardioverter defibrillators (ICDs) are not always needed to deliver the therapy [5].

A number of published economic studies have looked at the cost-effectiveness of the CRT-P and CRT-D devices for patients with CHF. The studies have mostly shown that the incremental cost-effectiveness ratios (ICERs) of CRT-D compared to optimal pharmacological therapy (OPT) alone or in combination with a CRT-P were too high due to large numbers of patients not requiring shock therapy [8, 9]. The main aim of the present study was to critically review economic models evaluating CRT-D devices for patients with heart failure (HF), compare the structure and inputs of the cost-effectiveness models, and identify the main factors influencing the cost-effectiveness of CRT-D devices in comparison to OPT alone or in combination with CRT-P.

## Methods

A systematic literature review was performed in order to identify the existing full health-economic models indexed in the main electronic databases such as Medline (Ovid), Embase (Ovid) and EconLit. The search was limited to articles published in the English language during the period from January 2000 to December 2014. The search strategies used a combination of Medical Subject Heading (MeSH) and free-text terms grouped into four categories; disease specific, device specific, economics and type of study. The relevant MeSH terms included: ‘cardiac pacing, artificial’, ‘pacemaker, artificial’, ‘heart-assist devices’, ‘heart conduction system’, ‘defibrillators, implantable’, ‘costs and cost analysis’, ‘economics, hospital’, ‘economics, medical’, ‘economics, nursing’, ‘economics, pharmaceutical’, ‘cost-effectiveness’, ‘humans’. References of the identified articles were scrutinized for additional references. Search strategies used to retrieve references from Medline (Ovid) and Embase (Ovid) are given in Annex 1.

The selection of the studies was done through pre-developed inclusion criteria. Only model-based economic evaluations (studies that included decision-tree models and Markov chain models) of implantable CRT-D devices were included. Trial-based economic evaluations were not eligible since most clinical studies have a short follow-up period while benefits of the CRT-D devices are not fully observed until the long term, and therefore we considered predictive modelling as a more valid approach to capture all costs and benefits of the therapy. All studies that were outside of the review scope, i.e. economic evaluations alongside clinical trials, reviews, meta-analyses, editorials, resource use studies or studies on costs were excluded. The first author performed the search and initial classification of the retrieved articles. All the selected articles were read independently by two reviewers (FT, ADIvA) and only those fulfilling the selection criteria were included in the review. Data extraction included: authors, year of publication, type of study and analysis, country of analysis, model structure, sources of effectiveness data, sources of economic data, sources of health state utilities, main comparators, outcomes and perspective taken as well as the main findings.

## Results

### Search results

**Fig. 1:**
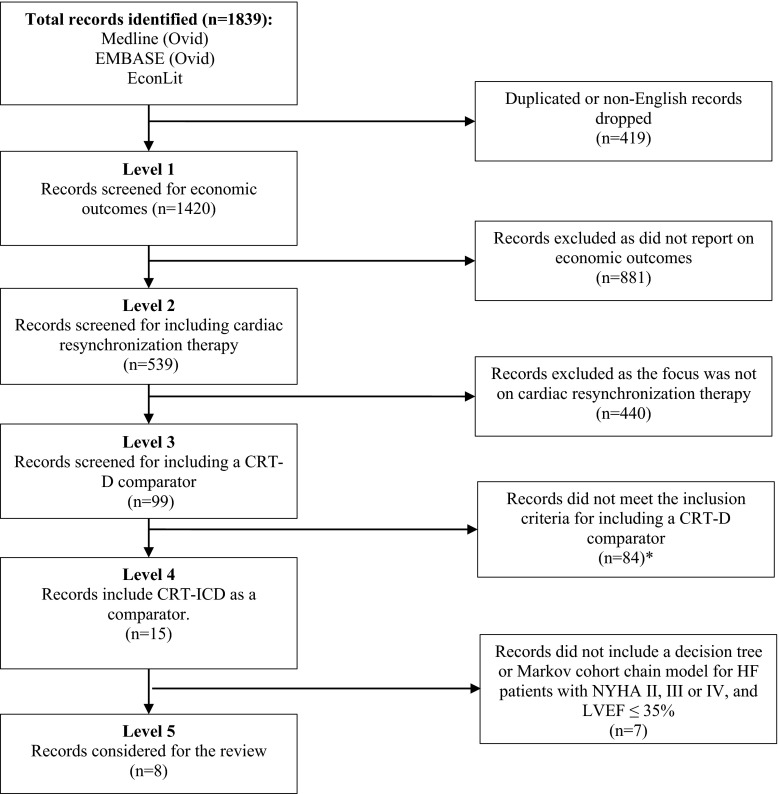
The selection process *Reviews, editorials, resource use and cost studies, as well as economic evaluations alongside a clinical trial were excluded at this step. *CRT-D*, cardiac resynchronization therapy device with the addition of an implantable cardioverter-defibrillator; *HF,* heart failure; *NYHA,* New York Heart Association functional classification; *LVEF,* left ventricular ejection fraction

The search retrieved 1839 citations, which were reduced to 1420 after excluding for duplicates and for non-English language citations. After screening titles and abstracts, 99 articles (Fig. 1), were further scrutinized to exclude papers that fell outside the scope of the review. The remaining references (15 studies) were scrutinized to include only studies on: (1) HF patients with NYHA II, III or IV, LVEF ≤ 35 % [10], (2) treated with the CRT-D as a comparator and (3) that included decision-tree models or Markov chain models. The eight studies remaining [11–18] included model-based economic evaluations (i.e., Markov models and decision-tree models) of CRT-D implantations and were included for further review.

Table 1 gives the general study characteristics of the selected publications for this review. The selected studies included economic models adapting perspectives of four European health care systems (Belgium, Germany, Spain and UK) [11–15, 17, 18] as well that of a middle income country (Brazil) [16].

**Table 1:** Overview of the selected papers

Author	Comparators	Perspective	Model structure	Analysis type	Time horizon (base case analysis)	Primary outcome	Target population	Source of effectiveness data	Source of economic data and price year	Sensitivity analysis
Fox et al. [11]	CRT-D vs OPT CRT-P vs OPT CRT-D vs CRT-P	Payer (United Kingdom, NHS)	Markov model	CEA and CUA	Lifetime horizon	Life years QALYs	HF patients with LVSD (EF < 35 %) NYHA class III or IV and QRS interval > 120 ms	CARE-HF [2] (CRT-P vs OPT)	UK NHS, literature and expert opinion (2006)	Univariate sensitivity on key variables: probabilistic sensitivity analysis
Yao et al. [12]	CRT-D vs OPT CRT-P vs OPT CRT-D vs CRT-P	Payer (UK NHS)	Markov model	CEA and CUA	Lifetime horizon	Life years QALYs	HF patients with LVSD (EF < 35 %) NYHA class II, III or IV LVEF of 30 mm (indexed to height), QRS interval > 120 ms	CARE-HF [2] (CRT-P vs OPT)	UK NHS, 2005	Univariate sensitivity on all variables: probabilistic sensitivity analysis
Aidelsburger et al. [13]	CRT-D vs OPT	Payer (German HCS)	Decision tree and Markov model	CEA and CUA	2 years	Life years QALYs and hospitalisations	HF patients with NYHA class III or IV	COMPANION [1] (CRT-P vs CRT-D)	German HCS, 2005	Univariate sensitivity on key variables: probabilistic sensitivity analysis not reported
Bond et al. [14]	CRT-D vs OPT CRT-D vs CRT-P	Payer (United Kingdom, NHS)	Markov model	CUA	Lifetime horizon	QALYs	HF patients with LVSD (EF < 35 %) NYHA class III or IV and QRS interval > 120 ms	CARE-HF [2]		Univariate sensitivity on key variables: probabilistic sensitivity analysis
Callejo et al. [15]	OPT vs CRT-P vs CRT-D	Payer (Spanish, PHCS)	Decision tree and Markov model	CEA and CUA	Lifetime horizon	Life years QALYs	HF patients with NYHA class II, III or IV and prolonged QRS	CARE-HF [2] (CRT-P vs OPT)	Spanish HCS, 2009	Univariate sensitivity on all variables: probabilistic sensitivity analysis
Bertoldi et al. [16]	CRT-D vs OPT CRT-P vs OPT ICD vs OPT CRT-D vs CRT-P CRT-D vs ICD	Payer (Brazilian HCS)	Decision tree and Markov model	CEA and CUA	20 years	Life years QALYs	HF patients with LVSD (EF < 35 %) NYHA class II, III or IV prolonged QRS	Meta-analysis	Brazilian HCS, 2011	Univariate sensitivity on all variables: probabilistic sensitivity analysis
Neyt et al. [17]	CRT-P vs OPT CRT-D vs CRT-P	Payer (Belgian HCS)	Decision tree and Markov model	CEA and CUA	Lifetime horizon	Life years QALYs	HF patients with LVSD (EF ≤ 35 %) NYHA class III or IV prolonged QRS	COMPANION [1] (CRT-P vs CRT-D)	Belgian HCS, 2010	No univariate sensitivity results reported; Scenario analysis on mortalities, hospitalisations, discount rates and device service life: probabilistic sensitivity analysis
Colquitt et al. [18]	CRT-D vs OPT CRT-P vs OPT CRT-D vs CRT-P CRT-D vs ICD	Payer (United Kingdom, NHS)	Markov model	CEA and CUA	Lifetime horizon	Life years QALYs	HF patients with reduced LVSD and cardiac dyssynchrony despite OPT Patients at increased risk of SCD as a result of ventricular arrhythmias despite receiving OPT	CARE-HF [2] (CRT-P vs OPT); MADIT-CRT [21] (CRT-D vs ICD); RAFT (CRT-D vs ICD)	UK NHS, literature and expert opinion (2013)	Univariate sensitivity on key variables: probabilistic sensitivity analysis

*CRT-P* implantable cardiac resynchronization therapy device with a biventricular pacemaker, *CRT-D* cardiac resynchronization therapy device with the addition of an implantable cardioverter-defibrillator, *OPT* optimal drug therapy, *ICD* implantable cardioverter-defibrillator, *NHS* National Health Service, *HCS* health care system, *PHCS* public health care system, *CEA* cost-effectiveness analysis, *CUA* cost utility analysis, *QALYs* quality of life adjusted life years, *HF* heart failure, *LVSD* left ventricular systolic dysfunction, *NYHA* New York Heart Association functional classification, *SCD* sudden cardiac death, *LVEF* left ventricular ejection fraction

### Modelling approaches

All models distinguish between a short-term (represented by costs and consequences of the process of device implantation) and a long-term phase (represented by the costs and consequences of the post implementation follow-up period). The initial short-term implantation phase was generally 4 weeks while the long-term maintenance phase was the lifetime of the patient (see Table 1). Four out of the eight selected studies [13, 15–17] used a decision tree to model the short-term phase while four of the other studies [11, 12, 14, 18] included this as an initial phase into the Markov cohort models. All the selected studies [11–18] used a Markov cohort model for the long-term maintenance period.

Paths in the short-term decision trees consisted of combinations of four treatment strategies: (1) optimal medical therapy (OPT), (2) CRT-P, (3) CRT-D and (4) implantable cardioverter defibrillator (ICD). Bertoldi et al. [16] linked successful implantation of the device (CRT-P, CRT-D or ICD) to complications or no-complications after implantation, which then led to the long-term model. Failure of implantation led to the long-term OPT strategy. This was similar to Yao et al. [12] except that these investigators allowed for up to three re-implantation attempts.

Markov states used in the eight selected studies [11–18] can be broadly classified into four categories: (1) the short-term implantation period, (2) upgrading or switching between different implantable devices (e.g. switching from CRT-P to CRT-D), (3) the maintenance states after device implantation or when on OPT therapy; and (4) the death state. Table 2 gives a more detailed summary of these Markov states.

**Table 2:** Overview of the Markov states in the selected studies

Health state categories in the Markov cohort models	Fox et al. [11]	Yao et al. [12]	Aidelsburger et al. [13]	Bond et al. [14]	Callejo et al. [15]	Bertoldi et al. [16]	Neyt et al. [17]	Colquitt et al. [18]
*Short-term states*								
CRT-P implantation (operation)	√	n.a.	n.a.	√	√	n.a.	√	√
Complications after CRT implantation	√	n.a.		√	√	n.a.		√
Death (CRT-P implantation or subsequent operation)	√	n.a.	n.a.	√	√	n.a.	√	√
*Upgrading/switching state*								
Upgrading CRT-P to a CRT-D (operation)	√		n.a.	√	√		√	√
ICD implantation (operation)	√	n.a.	n.a.	n.a.	n.a.	n.a.	√	√
*Maintenance states*								
Patient with CRT has no adverse events^a^	√	√	√	√	√	√	√	√
Patient receiving OPT has no adverse events^a^	√	√	√	√	√	√	√	√
Patient with ICD has no adverse events^a^	√	n.a.	n.a.	n.a.	n.a.	√	√	√
Hospitalization—CRT-related infection	√	√	√		√	√	√	√
Hospitalization—ICD-related infection^b^	√		n.a.	n.a.	n.a.	√		√
(Hospitalization)—lead failure/displacement	√		√		√	√		√
HF hospitalisation	√		√	√	√	√		√
Hospitalisation—heart transplant	√	√	√					√
Hospitalisation—CABG		√	√					
Hospitalisation—Radiofrequency ablation		√	√					
Hospitalisation—PTCA/Stent		√	√					
Maintenance of CRT (e.g. device/battery change) (operation)	√			√				√
*Long-term death states*								
Death from SDC	√	√	√	√	√	√	√	√
Death from HF	√		√		√	√		√
Death from nCR causes	√		√		√			√

^a^This means that these events do not take place during the model cycle

^b^Yao et al. [12] include two additional states the Coronary Care Unit (CCU) and the intensive care unit (ICU)

*SDC* Sudden Cardiac Death, *HF* Heart failure, *nCR* non-cardiac-related, *PTCA* Percutaneous Transluminal Coronary Angioplasty, *n.a.* not applicable

As mentioned, four out of the eight delected studies [11, 12, 18] included the initial phase of the device implementation in the Markov model by adding the following states: (1) surgical intervention for CRT implantation, (2) hospitalisation because of complications after CRT implantation and (3) death because of CRT implantation (or any subsequent operation). The consequent maintenance states usually referred to events like stable state (while on CRT or OPT), hospitalisation due to lead displacements or infections, hospitalisation due to HF worsening and hospitalisation due to other causes (e.g. heart transplant, CABG, ablation, etc).

All models allowed patients to move from one Markov state to another. Death states were detailed in SCD, HF death, or death from non-cardiac related (nCR) causes. In Yao et al. [12] patients that remained alive in the next time period could continue being in the same NYHA class or move to higher or lower NYHA classes. Similarly, in Bertoldi et al. [16] patients could move between NYHA classes (but with only a NHYA class at a time).

Upgrading devices from CRT-P to CRT-D was allowed in three of the selected studies [11, 15, 17]. In such cases CRT-P patients could at any time experience arrhythmia and thereafter upgrade to a CRT-D device. In addition to the above states the model by Fox et al. [11] (which consists of a synthesis of the existing models) allowed also for device explanting or replacement of the CRT-D device with a new one. Patients could also be switched to the OPT arm or get an ICD alone but only when experiencing arrhythmias or receiving a heart transplant (heart transplant state was also allowed in Colquitt [18]).

### Patient population

The population considered in the selected studies were adults (aged 18 and over) eligible for CRT implantation. Eligibility criteria in all the studies were largely associated with the guidelines for CRT implantation in patients suffering from heart failure. These guidelines recommend implantation of a CRT device (with or without ICD) for patients who have left ventricular ejection fraction (LVEF) ≤35 %, a QRS duration ≥120 ms, sinus rhythm, and fall within NYHA functional class III or ambulatory class IV heart failure [4, 10, 19, 20]. Most of the studies complied with these criteria. Five out of the eight selected studies [11, 12, 14, 15, 18] used clinical data from the Care-HF trial, (a multicentre, international randomized trial comparing CRT-P to OPT) [2], while two other studies [13, 17] used data from the COMPANION trial (also a multicentre, international randomized trial comparing CRT-P to OPT and CRT-D to OPT) [1]. Both trials used similar eligibility criteria for the selection of patients [1, 2]. Bertoldi et al. [16] used data from an outpatient clinic in a Brazilian hospital but reported a similar target population. Colquitt et al. [18] used data from the MADIT-CRT [21] and RAFT [22] trials for the comparison of CRT-D with ICD arms. Colquitt et al. [18] distinguished between three different population groups. However, for consistency with the populations of other studies, here we focus on the analysis of group II (i.e., patients with heart failure as a result of left ventricular systolic dysfunction (LVSD) and cardiac dyssynchrony despite receiving OPT) [18].

### Model comparators

Six [11, 12, 14–17] out of eight selected studies included three comparators; CRT-P with OPT, CRT-D with OPT, and OPT alone. One study [13] compared only cost-effectiveness of CRT-D against CRT-P. Colquitt et al. [18] included also comparisons of ICD with OPT and CRT-D (though this was done in a different patient population).

### Model time horizon

Pharmacoeconomic guidelines agree that the time horizon of a cost-effectiveness model should extend far enough in the future to capture the major health and economic outcomes, including both the intended and unintended effects [23]. With treatment for heart failure, certain treatment outcomes can be realized over a shorter period (like the outcome of the surgical intervention) while others, such as a possible effect on survival, can only be realized over a long time horizon (extending to lifetime of the patient). The duration of clinical trials testing the cardiac resynchronization therapy in heart failure disease varies from 6 months [21, 24] to 12 months [25] or to a longer period of 29 months [2]. In the selected modelling studies, patient outcomes and costs were simulated over 20 years [16] or over the complete lifetime of the patient [11, 12, 14, 15, 17, 18], except for one study [13] which applied a time horizon of only 2 years after implantation (Table 1). This is considered rather a conservative approach, as the high costs of implantation cannot be fully recovered within such a short term. The authors justified their choice by explaining the difficulties in the extrapolation of utilities, costs and transition probabilities beyond the 2-year follow-up of the COMPANION trial [1].

### Resource use and unit prices

Resource use and unit prices in the selected studies were predominantly obtained from the health care systems of the respective countries. All the eight selected studies [11–18] employed a payer perspective. As such, they have included costs of CRT-P, CRT-D and ICD devices, costs of device implantation, lead replacement, heart failure hospitalisation and follow-up costs while in a stable health state. The level of detail varied substantially between the studies, and was predominantly dependent on the structure of the short-term or long-term models (see also above).

It should be noted that prices of devices differed between studies reflecting also the market value of the devices over time. Hence, earlier studies by Fox et al. and Yao et al. [11, 12] estimated prices of the CRT-D devices in the UK healthcare setting at respectively €19,196 and €19,914 (converted into 2014 prices in euros using OECD estimates of purchasing power parities (PPPs) for GDP [26] while the most recent study from Colquitt et al. [18] estimated this at only €13,170 (PPP adjusted and in 2014 prices).

### Health state utilities

The primary outcomes were quality-adjusted life years (QALYs) or life years (LYs). All the studies use the NYHA Functional Classification to assign the quality of life (QoL) scores for patients in each health state. Again, the only exception here is the study by Aidelsburger et al. [13] that also included hospitalisations as an outcome.

The calculation of QALYs in the selected studies was based on utility values from NYHA classes (Table 3). Seven out of eight studies [11–16, 18] distinguish between utility values for each NYHA class. Neyt et al. [17] considered mean health utilities by treatment rather than by NYHA class. They argued that this was preferred, given the substantial variation of NYHA class utility estimates between publications. In fact, health utility values used per NYHA class do vary greatly between studies, as can be seen from values in Table 3.

**Table 3:** Values of health state utilities for the selected studies

	Mean Utility	95 % CI	Min–max values	Source
Yao et al. [12] and Aidelsburger et al. [13]				
NYHA class I	0.82	(0.78:0.85)		[2]
NYHA class II	0.72	(0.69:0.75)		[2]
NYHA class III	0.59	(0.55:0.63)		[2]
NYHA class IV	0.51	(0.41:0.61)		[2]
Fox et al. [11] and Bond et al. [14]				
NYHA class I	0.93		(0.91:0.96)	[8]
NYHA class II	0.78		(0.72:0.84)	[8]
NYHA class III	0.61		(0.59:0.63)	[27]
NYHA class IV	0.44		(0.42:0.46)	[27]
Bertoldi et al. [16]				
NYHA class I	0.90		(0.71:0.94)	[28–30]
NYHA class II	0.83		(0.61:0.94)	[28–30]
NYHA class III	0.74		(0.52:0.84)	[28–30]
NYHA class IV	0.60		(0.42:0.74)	[28–30]
Callejo et al. [15]				
NYHA class I	0.69		(0.53; 0.85)	[31]
NYHA class II	0.60		(0.46; 0.74)	[31]
NYHA class III	0.49		(0.34; 0.64)	[31]
NYHA class IV	0.35		(0.15; 0.55)	[31]
Neyt et al. [17]^a^	0.78	(0.73:0.83)^b^		[9]
Colquitt et al. [18]				
NYHA class I	0.86		(0.85:0.86)	[29]
NYHA class II	0.77		(0.76:0.78)	[29]
NYHA class III	0.67		(0.73:0.77)	[29]
NYHA class IV	0.53		(0.48:0.58)	

^a^Neyt et al. [17] use only mean utility values for the overall sample

^b^97.5 % confidence interval

### Cost-effectiveness results

ICERs for cost per QALY gained and cost per LY gained are presented in Tables 4 and 5. After checking for transferability criteria [32, 33], all ICERs were converted in 2014 prices in euros using PPPs [26]. Seven out of the eight selected studies [11, 12, 14–18] reported cost-effectiveness results per QALY for CRT-P compared to OPT and for CRT-D compared to CRT-P, four studies [11–13, 18] for CRT-D compared to OPT, and two studies [16, 18] for CRT-D compared to ICD (Table 4). Despite the differences in ICERs, results were consistent in showing that CRT-P was mostly cost-effective in comparison to OPT alone. However, results were less clear for ICERs of CRT-D compared to OPT. In the study by Aidelsburger et al. [13] this ICER was much higher than in other studies (€76,350) which could relate to the time horizon of only 2 years in this study. In general, ICERs for CRT-D compared to CRT-P were considerably higher than for CRT-D compared to OPT. Hence, ICERs for CRT-D versus CRT-P ranged from €42,986 to €63,343 while ICERs per QALY for CRT-D versus OPT ranged from €16,166 to €29,889. The only exception was the study from Colquitt et al. [18] where both ICERs were comparable (€29,889 vs €30,447), but these did not apply to the same population [18]. It should be noted that most ICERs for CRT-D versus CRT-P are well above what is considered cost-effective in most countries (for instance the £20,000-£30,000 threshold applied in the UK [34]—converted to €21,427-€32,139 PPP adjusted). Bertoldi et al. [16] suggested that CRT-D therapy should not be systematically recommended for CRT-P eligible patients, while it can be an option for ICD eligible patients. Yao et al. [12] suggested that CRT-D was not cost-effective, particularly for CRT-P patients with poor life expectancy. Fox et al. [11] called for more research to explore the added value of CRT-D over CRT-P and for improving identification of non-responders among patients in the CRT-D group.

**Table 4:** Incremental cost-effectiveness ratios (in euros per QALYs gained)^a^

	CRT-P versus OPT	CRT-D versus OPT	CRT-D versus CRT-P	CRT-D versus ICD
Fox et al. [11]	€20,077	€28,372	€48,179	–
Yao et al. [12]	€6763	€16,166	€42,986	–
Aidelsburger et al. [13]	–	€76,350	–	–
Bond et al. [14]	€19,865	–	€47,662	–
Callejo et al. [15]	€30,307	–	€56,719	–
Bertoldi et al. [16]	€11,808	–	€63,343	€32,664
Neyt et al. [17]	€9849	–	€49,774	–
Colquitt et al. [18]	€29,551^b^	€29,889^a^	€30,447^a^	€29,135^b^

^a^Indexed for purchasing power parities for GDP [26] and in 2014 prices

^b^Corresponds to population II in Colquitt et al. [18], i.e., patients with heart failure as a result of LVSD and cardiac dyssynchrony despite receiving OPT; Corresponds to population III in Colquitt et al. [18] i.e., group II plus patients at risk of SDC due to ventricular arrhythmias despite receiving OPT)

**Table 5:** Incremental cost-effectiveness ratios (International € per LYs gained)^a^

	CRT-P versus OPT	CRT-D versus OPT	CRT-D versus CRT-P	CRT-D versus ICD
Fox et al. [11]	–	–	–	–
Yao et al. [12]	€6291	€32,179	€32,179	–
Aidelsburger et al. [13]	–	€168,040	–	–
Bond et al. [14]	–	–	–	–
Callejo et al. [15]	€24,806	–	€34,160	–
Bertoldi et al. [16]	€22,088	–	€46,890	€34,054
Neyt et al. [17]	€11,256	–	€38,781	–
Colquitt et al. [18]	€31,060^b^	€13,926^b^	€7375^b^	€21,411^b^

^a^Indexed for purchasing power parities for GDP [26] and in 2014 prices

^b^Corresponds to population II in Colquitt et al. [18], i.e., patients with heart failure as a result of LVSD and cardiac dyssynchrony despite receiving OPT; Corresponds to population III in Colquitt et al. [18] i.e., population group II in (a) plus patients at risk of SDC due to ventricular arrhythmias despite receiving OPT)

Two studies [16, 18] reported ICERs for QALYs gained for CRT-D compared to ICD. Both ICERs were comparable, ranging from €29,135 to €32,664 (even though they concern different populations). Colquitt et al. [18] found such ICERs to be robust and influenced only from all-cause mortality in the ICD-only arm and lifetime of CRT-D and ICD devices [18].

Two of the selected studies [11, 14] did not report on ICERs per LY (Table 5). As for the other studies, similar trends held as in ICERs per QALY. The ICER for the comparison between CRT-D and OPT in the Aidelsburger et al. study [13] appears to be much higher than others, while ICERs for CRT-D against CRT-P remain constantly higher than ICERs for CRT-D against OPT. Again the study from Colquitt et al. [18] is an exception here, although these results should be cautiously interpreted as the population in Colquitt et al. [18] is not the same as in other studies.

### Uncertainty

All selected studies [11–18] reported univariate sensitivity analysis on key variables. Key determinant variables were battery longevity [12, 13, 16, 18], cost of the device [16, 18] as well as relative risk for mortality from HF (for CRT-P vs OPT or CRT-D vs CRT-P) [14, 16, 18]. Decreasing the cost of the CRT device by 50 % decreased the ICERs by 23 % for CRT-D vs CRT-P or by 40 % for CRT-P vs OPT [16]. The selected studies assumed a base case battery life that varied from 5 [16] to 6.5 years [14, 15] for CRT-P devices and from 5 [16, 17] to 5.5 years [14, 15] for CRT-D. Yao et al. [12] assumed a base case battery life of 7 years for CRT-D. Increasing battery life by 40 % decreased the ICERs by more than 20 % in Bertoldi et al. [16] and by 29 % in Colquitt et al. [18]. The reduction of mortality with CRT-D by 13.3 % decreased the ICER for CRT-D vs CRT-P by 36 % [16] while the decrease in relative risk for HF death in CRT-D patients by 20 % decreased the ICER by 58 % [15]. Five studies reported probabilistic sensitivity analyses [11, 12, 16–18], while one study [13] stated to have performed a two-way sensitivity analysis, but did not report the results. Four out of the eight selected studies [11, 12, 17, 18] incorporated cost-effectiveness acceptability curves (CEACs), which are used to summarize the uncertainty in the cost-effectiveness estimates. The CEACs in these four studies showed that, for a willingness-to-pay (WTP) threshold between €27.000 and €44.000 (PPP adjusted) per QALY, the probability of CRT-D being cost-effective compared to OPT or CRT-P was only 26–40 %.

## Discussion

The review of the 6 selected economic evaluation models from January 2000 to December 2015 showed that CRT-P devices for HF patients could be considered a cost-effective therapy, if compared to OPT. However, implanting a CRT-D device instead of a CRT-P appeared much less cost-effective. Sensitivity analysis showed that cost-effectiveness of CRT-D over CRT-P depends on costs of device, battery life and relative risk for HF death in CRT-P patients. Most of the selected studies agreed that there is a need for a better identification of patients that will have a substantially improved life expectancy after implanting the CRT-D [11, 14, 15, 17].

The review showed that incremental cost-effectiveness ratios depend on characteristics of the models adopted by the selected studies. We have identified some variability in the decision models used by the selected studies. Such variability was observed around a number of methodological domains and the main assumptions used. First, the modelling approaches included both decision-trees and Markov structures. In chronic diseases Markov models are preferred over decision trees as the latter ones can get too complex over a longer time span, if they should account for switching between health states. Instead, Markov models can be more flexible and able to incorporate a series of transitions between health states over a number of discrete time periods [35–37]. However, the combination of both short-term decision trees and Markov structures in four of the selected studies allowed the different treatment strategies and various complications (associated with the implantation period) to be captured in the model. In fact, such a combination seems to be common practice in economic models of CRT for HF [11] and it is very unlikely to impact the ICERs.

We have also found that numbers of health states in the Markov structures varied between the selected studies, especially regarding the hospitalisation states. It is usually recommended that the number of states is kept as small as possible given that estimations of deterministic models using averages can cause statistical bias in average outputs [40, 41]. However, there are no reasons to believe that such differences could have been main causes behind the differences in ICERs in our selected models.

Other modelling differences in the selected studies included treatment cross-overs, (i.e. upgrading from CRT-P to CRT-D [11, 13–15, 17] or downgrading from CRT-D to ICD [11]) and using utility values per NYHA class [11, 12, 14–16, 18] versus the mean utility over all NYHA classes [17]. However, it is difficult to speculate on the impact these differences may have had on the ICERs.

The assumptions on the HF mortality and the hospitalisation rates of patients having a CRT-D as compared to those staying on OPT or having CRT-P alone were considered important in accurately simulating real-life events. All the selected studies in this review used data from existing clinical trials [1, 2, 21] whose follow-up periods were much lower than the time horizon chosen in the studies. The incremental effectiveness of CRT-D after the follow-up period of the trials was maintained constant over time in all the selected studies [11–18]. This may have potentially led to an overestimation of the incremental effectiveness of CRT-D as it is likely that relative benefits of CRT-D fade out as severity of HF increases.

The assumed battery life of the pulse-generating devices was also shown to be an important determinant of the uncertainty in the cost-effectiveness estimates. The service time of the CRT-P and CRT-D devices is limited by the battery life (as battery replacement alone is not feasible and a surgical operation is needed for the replacement of the entire unit) [18]. An assumption of longer intervals for device replacement would make the CRT-D appear more cost-effective than the comparators. We found that the studies did not differ very much on this assumption, though longer service times could potentially increase the cost-effectiveness of CRT-D in the future.

Additional sources of variability were differences in resource use and unit prices across the studies, differences in time horizons applied, and uncertainty around the primary efficacy of data used in the models. Resource use and unit prices may create difficulties in comparison of the results across jurisdictions [38]. Therefore, any comparison of the results in this review should be considered with caution. On the other hand, as shown here, the costs of the CRT devices tended to decrease over time even within the same country [11, 12, 18] and this can be a crucial factor in determining cost-effectiveness in the future.

The time horizon applied is also important as the full effects of CRT on patient survival can only be revealed over a patient’s lifetime [39]. Aidelsburger et al. [13] had a much shorter life horizon, which directly impacted the ICERs of CRT-D in comparison with CRT-P. The uncertainty around the efficacy data used in the models (derived from different clinical trials or meta-analyses) hampers the interpretation of results. There was only one head-to-head comparison trial for CRT-P versus CRT-D [25]. However, this trial supported only the advantages of CRT-P over OPT and CRT-D over OPT. There is no broad consensus on the advantages of CRT-D over CRT-P, even though a meta-analysis showed some superiority of the former on all-cause death rate and cardiac death after 1-year follow-up [3]. Other studies pointed out that CRT-D may be especially beneficial to a particular group of patients, like women, those with longer QRS duration, and smaller baseline LV volumes [42]. This review noted that the ICERs for CRT-D versus CRT-P are still above what most countries are willing to pay for an additional QALY (e.g. the £20,000-£30,000 per QALY threshold applied in the UK [34]). It is sensible to believe that a better identification of patients for whom this technology is beneficial would reduce unavoidable costs by making CRT-D a more cost-effective alternative.

## Conclusions

The studies included in this review seem to converge around the finding that while CRT-P and CRT-D can be considered cost-effective if compared to OPT, cost-effectiveness of CRT-D over CRT-P remains questionable. There is no broad consensus of the relative effectiveness of CRT-D over CRT-P, and therefore studies looking at the all-cause death rate and HF death rate could prove to be important in reducing the uncertainty around cost-effectiveness results. In addition, given the high proportion of eligible patients not responding to CRT [7] or not needing the addition of an ICD [5], future studies need to better identify CRT-P patients that will have a reasonable life expectancy when treated with CRT-D. This would bring down avoidable costs, and consequently improve cost-effectiveness of CRT-D over CRT-P.

## Appendix: Search strategy for cost-effectiveness studies

Medline (OvidSP) and Embase (OvidSP)

1. Cardiac pacing, artificial/
2. Pacemaker, artificial/
3. Heart-Assist Devices/
4. Heart Conduction System/
5. Defibrillators, implantable/
6. CRT-P.mp.
7. CRT-D.mp.
8. (CRT or “cardiac resynchron$ therap$”).ti,ab.
9. (resynchroni$ation or cardiac resynchronization therapy or biv).tw.
10. (biventricular adj2 (pacing or pacer or pacemaker or device)).tw.
11. ((implantable cardioverter-defibrillator) or (implantable cardioverter$ adj2 defibrillator$)).tw
12. (dual adj2 chamber adj2 (pacing or pacer or pacemaker)).tw.
13. (antitachycardia adj2 (pacing or pacemaker or pacer or device)).tw.
14. (implantable$ adj2 cardioverter and defibrillator$).ti,ab,ot,hw.
15. or/1-14
16. ((arrhythmia$) or (tachycardia$) adj2 (ventricular or fibrillation)). ti,ab,ot,hw.
17. (heart adj4 failure).mp.
18. (left adj2 ventricular adj2 function or dysfunction).tw.
19. (ventricular adj2 tachycardia$).tw.
20. Dyssynchrony.tw.
21. or/16-20
22. economics/
23. exp “costs and cost analysis”/
24. exp “economics, hospital”/
25. economics, medical/
26. economics, nursing/
27. economics, pharmaceutical/
28. (economic$ or cost or costs or costly or costing or price or prices or pricing or pharmacoeconomic$).ti,ab.
29. (expenditure$ not energy).ti,ab.
30. value for money.ti,ab.
31. budget$.ti,ab.
32. *or/22*-*31*
33. ((energy or oxygen) adj cost).ti,ab.
34. (metabolic adj cost).ti,ab.
35. ((energy or oxygen) adj expenditure).ti,ab.
36. *or/33*-*35*
37. *32 not 36*
38. letter.pt.
39. editorial.pt.
40. historical article.pt.
41. *or/38*-*40*
42. *37 not 41*
43. Animals/
44. Humans/
45. 43 not (43 and 44)
46. 42 not 45
47. (Markov adj5 model$).ti,ab,pt
48. (Cohort$ simulation).tw
49. (Cost-effectiveness) .ti,ab,pt
50. Cost-effectiveness/
51. (Cost adj2 utility).ti,ab,pt
52. (Cost adj2 benefit).ti,ab,pt
53. or/47-52
54. 15 or 21
55. 54 and 46
56. 55 and 53
57. Limit 56 to yr=2000-2014
